# Supplementary material for: Higher harmonics in planar Hall effect induced by cluster magnetic multipoles
Source: Nat Commun. 2022 Oct 30;13:6501. doi: 10.1038/s41467-022-34189-6 (PMC9618580; doi:10.1038/s41467-022-34189-6)
Supplement: Supplementary file 1 — Supplementary Information [file 41467_2022_34189_MOESM1_ESM.pdf]

Supplementary Materials for:

Higher harmonics in planar Hall effect induced by cluster magnetic multipoles

Jeongkeun Song[1,2], Taekoo Oh[1,2], Eun Kyo Ko[1,2], Ji Hye Lee[1,2], Woo Jin Kim[3,4], Yangyu Zhu[5], Bohm-Jung Yang[1,2], Yangyang Li[5]*, and Tae Won Noh[1,2]*

[1] *Department of Physics and Astronomy, Seoul National University, Seoul, 08826, Korea*

[2] *Center for Correlated Electron Systems, IBS, Seoul, 08826, Korea*

[3] *Stanford Institute for Materials and Energy Sciences, SLAC National Accelerator Laboratory, Menlo Park, CA 94025, United States.*

[4] *Department of Applied Physics, Stanford University, Stanford, CA 94305, United States.*

[5] *School of Physics, Shandong University, Jinan, 250100, China*

*Correspondence to: yangyang.li@sdu.edu.cn; twnoh@snu.ac.kr;

Table of contents

Note 1. Anomalous Hall effect in the fully strained $Nd_2Ir_2O_7$ thin film.

Note 2. Extraction and fast Fourier transform of the planar Hall effect in the $Nd_2Ir_2O_7$ thin film.

Note 3. Theoretical derivation of orthogonal magnetization from magnetic octupole ordering and its effect on the planar Hall effect.

Note 4. Anomalous planar Hall effect in the $Nd_2Ir_2O_7$ thin film.

References

Note 1: Anomalous Hall effect in fully-strained $Nd_2Ir_2O_7$ thin film.

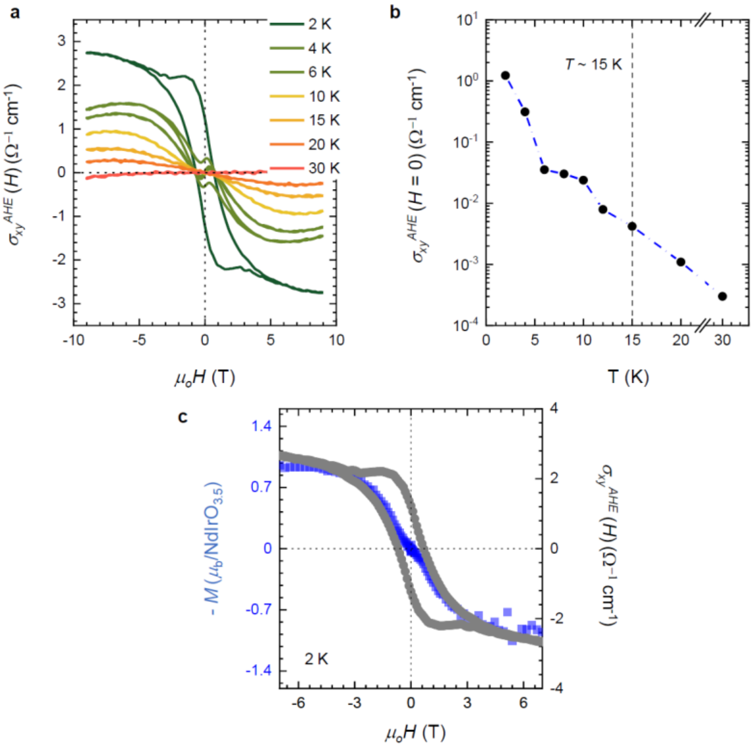

Figure s1 Anomalous Hall effect in the $Nd_2Ir_2O_7$ thin film. a. The anomalous Hall effect (AHE) was measured by applying H_{ext} along the [111] direction and a current along the $[1\bar{1}0]$ direction. **b.** Log scale plot of σ_{xy}^{AHE} (H = 0) vs. T. Note that below T ~ 15 K, σ_{xy}^{AHE} (H = 0) starts to increase due to the T_1-octupole. **c.** The magnetization curve at 2 K of the $Nd_2Ir_2O_7$ film indicates the absence of magnetization in the film.

In addition to crystallographic and resistivity characterization, characterization using the anomalous Hall effect (AHE) should be considered. Apart from domain walls[1], the strain-induced AHE is related to the existence of T_1-octupole ordering in this system[2]. Additionally, the hump-like feature of the NIO-227 film below 15 K is known as the *f*-*d* exchange interaction between Ir and the Nd lattice[2]. This hump-like feature can be fitted with two tanh functions attributed to Ir and Nd (Fig. s2). To confirm the existence of the T_1-octupole in the NIO-2227 thin film, we performed *T*-dependent AHE measurements (Fig. s1). Notably, the finite value of

σ_{xy}^{AHE} ($H = 0$) without magnetization starts to increase below 15 K (Fig. s1b and s1c), in which the T_1-octupole ordering induces this phenomenon[2].

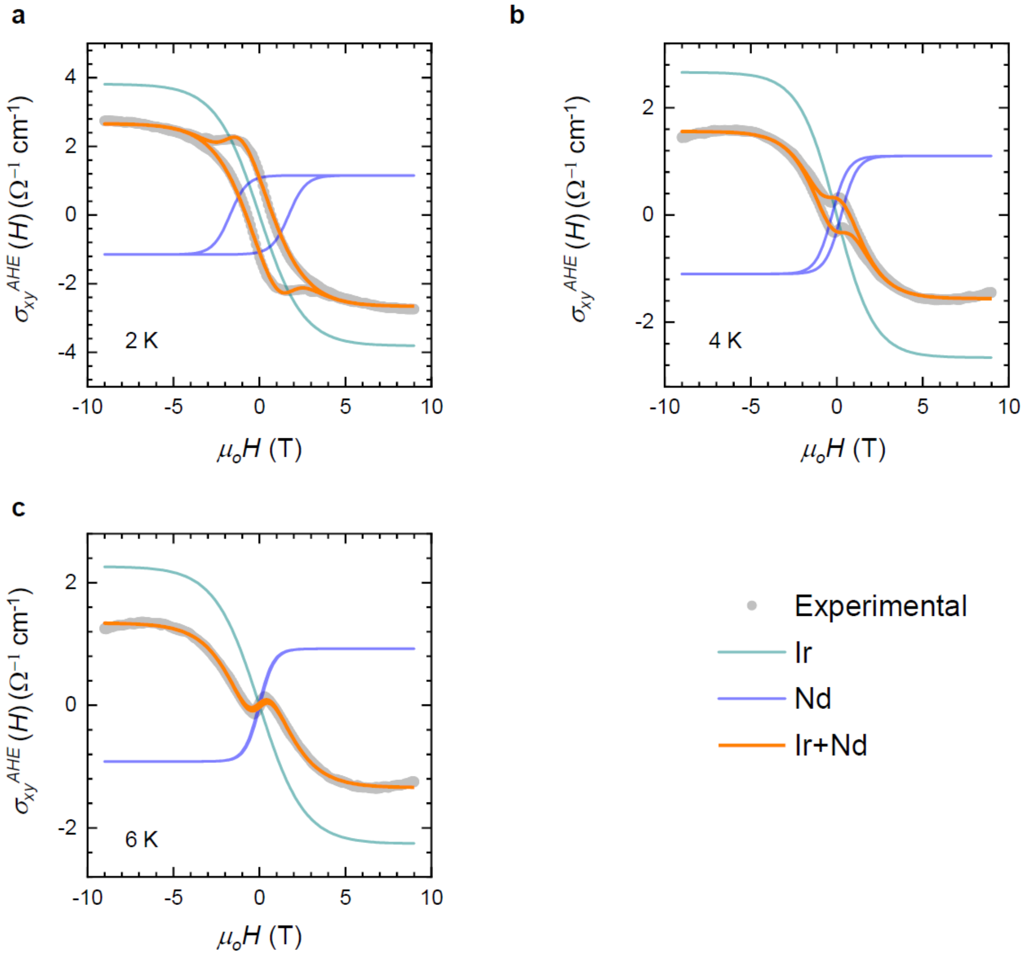

Figure s2. Fitting results for the AHE in fully-strained $Nd_2Ir_2O_7$ film. Experimental AHE data (grey circle) and fitting result with Ir (green) and Nd (blue) contribution at **a.** 2 K, **b.** 4 K, and **c.** 6 K.

The AHE from the octupolar order

Magnetic structures are closely linked to transport phenomena such as the AHE. In our strained $Nd_2Ir_2O_7$ thin films, the dipole, A_2-octupole, and T_1-octupole exist. The dipole is ferromagnetic order, which can induce the AHE. In contrast, the A_2-octupole and T_1-octupole are antiferromagnetic orders, in which AHE conventionally vanishes. However, we need to consider the crystalline symmetries to determine whether the octupoles are related to the AHE.

An easy way to determine whether antiferromagnetic order can induce the AHE is to compare its magnetic point group to that of ferromagnetic order. The AHE can be induced by breaking the time-reversal and crystalline symmetries by ferromagnetic order. For example, in the pyrochlore lattice, the point group is the T_d group, which includes 3-fold rotations about [111] and equivalent axes, 2-fold rotations about x, y, and z, diagonal mirrors, and 4-fold roto-inversions. Ferromagnetic order along one of the x-, y-, and z-axes breaks all symmetries except for 2-fold rotation about one axis and two 4-fold roto-inversions. Including antiunitary symmetries, the magnetic point group is $-42'\mathrm{m}'$. The symmetry breaking makes the AHE be induced in the plane perpendicular to the ferromagnetic axis.

Some antiferromagnetic orders can break the time-reversal symmetry, but this does not guarantee the appearance of the AHE. The A_2-octupole, for instance, breaks the time-reversal symmetry, but all 3-fold rotations and 2-fold rotations are still preserved. In particular, the magnetic point group is $-4'3\mathrm{m}'$, different from that of dipoles. Hence, the A_2-octupole contributes little to the AHE. In contrast, the T_1-octupole breaks the time-reversal and crystalline symmetries other than one 2-fold rotation and two 4-fold roto-inversions. The magnetic point group is $-42'\mathrm{m}'$, which is the same as that of dipoles. Therefore, the T_1-

octupole gives rise to the AHE similar to the dipole.

One can understand the argument based on the Hubbard Hamiltonian in the previous section. Let us think that strain and a magnetic field are absent. Without U, the energy band has a quadratic band crossing at the Gamma point. When U increases, the AIAO order develops, and the quadratic band crossing is broken into 4 pairs of Weyl nodes whose energy is exactly at the Fermi level. Each pair of Weyl nodes is at the Gamma-L line. In this case, the anomalous Hall conductivity is proportional to the distance between a pair of Weyl nodes. Since the sum of distances for all 4 pairs of Weyl nodes vanishes, the anomalous Hall conductivity vanishes. When U increases further, Weyl nodes meet at the L point and are annihilated by their counterparts. Then, the system becomes a trivial insulator, and the Hall conductivity again vanishes.

Note 2. Extraction and fast Fourier transform of the planar Hall effect in the $Nd_2Ir_2O_7$ thin film.

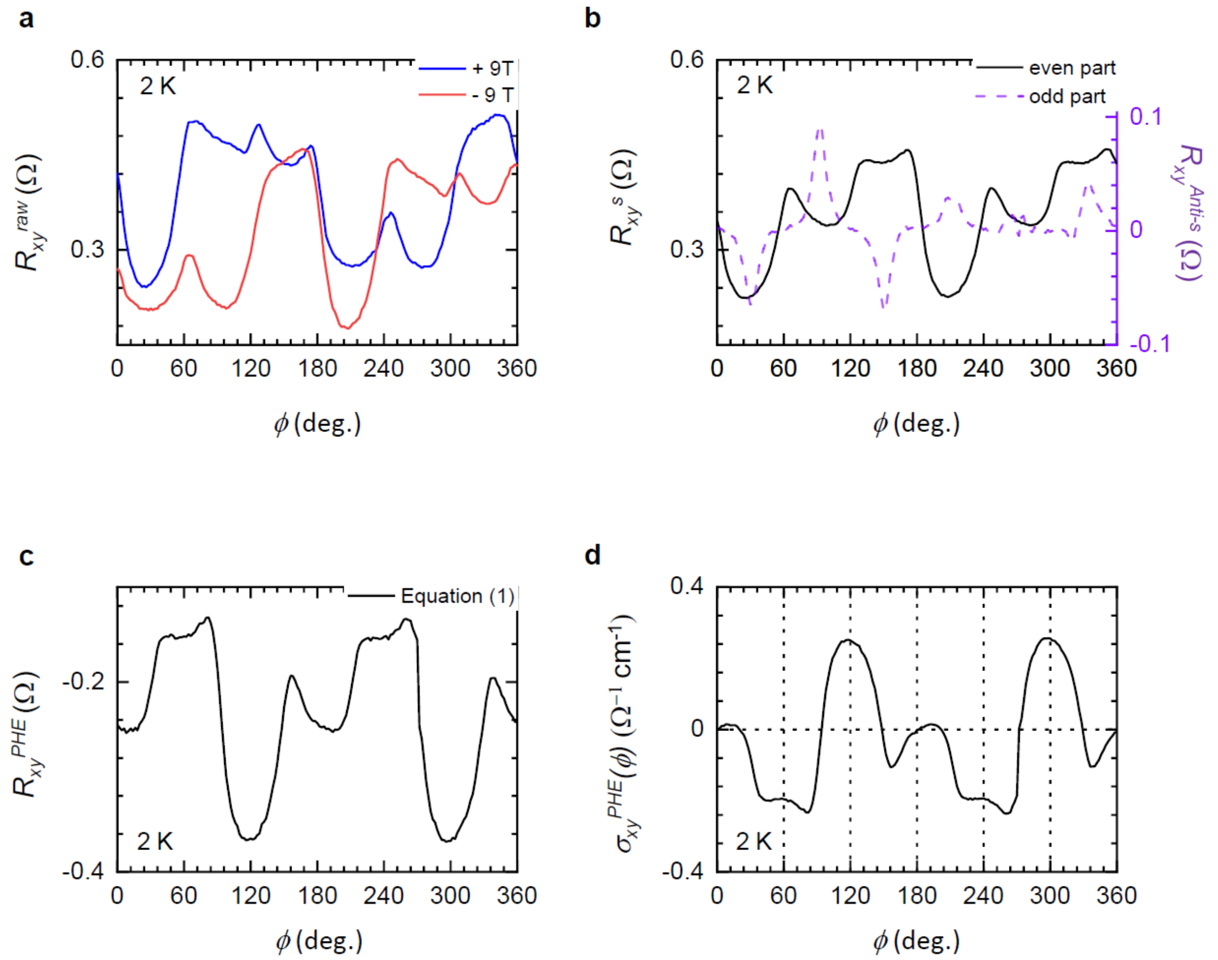

Figure s3 Symmetrization process of the planar Hall effect. The planar Hall effect was measured by applying a current along the $[1\bar{1}0]$ direction and rotating H_{ext} between $[11\bar{2}]$ and $[1\bar{1}0]$. The $T \sim 2$ K data are shown to demonstrate the process of extraction of the planar Hall effect. **a**. The raw data of $R_{xy}{}^{raw}$ were measured at $\pm$ 9 T with ϕ rotation. **b.** The even (odd) part of the $R_{xy}{}^{raw}$ curve was extracted by the symmetrization (antisymmetrization) equation $R_{xy}^{s}(\phi, H) = \frac{R_{xy}(\phi,\ H) + R_{xy}(\phi,\ -H)}{2}$ ($R_{xy}^{Anti-s}(\phi, H) = \frac{R_{xy}(\phi,\ H) - R_{xy}(\phi,\ -H)}{2}$). **c.** Plot of the R_{xy}^{PHE} curve. The contribution of longitudinal magnetoresistance was excluded by normalization using equation (1). **d.** Planar Hall conductivity σ_{xy}^{PHE} curve with respect to ϕ rotation obtained using $\sigma_{xy}^{PHE} = \frac{-\rho_{xy}^{PHE}}{\rho_{xy}^{PHE^2} + \rho_{xx}{}^2}$.

Unlike the AHE, where antisymmetrization is taken to exclude the possible longitudinal resistance contribution, the planar Hall effect is symmetrized and normalized. The first symmetrization is taken, as shown in Fig. s3b, by using $R_{xy}^{s}(\phi, H) = \frac{R_{xy}(\phi,\ H) + R_{xy}(\phi,\ -H)}{2}$. Then, $R_{xy}^{s}(\phi, H)$ is normalized to exclude the possible longitudinal resistance contribution using the equation from ref. 3:

$$R_{xy}^{PHE}(\phi, H) = R_{xy}^{s}(\phi, H) - R_{xx}(\phi, H)\frac{R_{xy}^{s}(\phi,\ H=0)}{R_{xx}(\phi,\ H=0)}, \quad (1)$$

Then, the planar Hall conductivity σ_{xy}^{PHE} is calculated by $\sigma_{xy}^{PHE} = \frac{-\rho_{xy}^{PHE}}{{\rho_{xy}^{PHE}}^2 + {\rho_{xx}}^2}$; here, $-\rho_{xy}^{PHE}\ and\ \rho_{xx}$ are the planar Hall effect resistivity and longitudinal magnetoresistance.

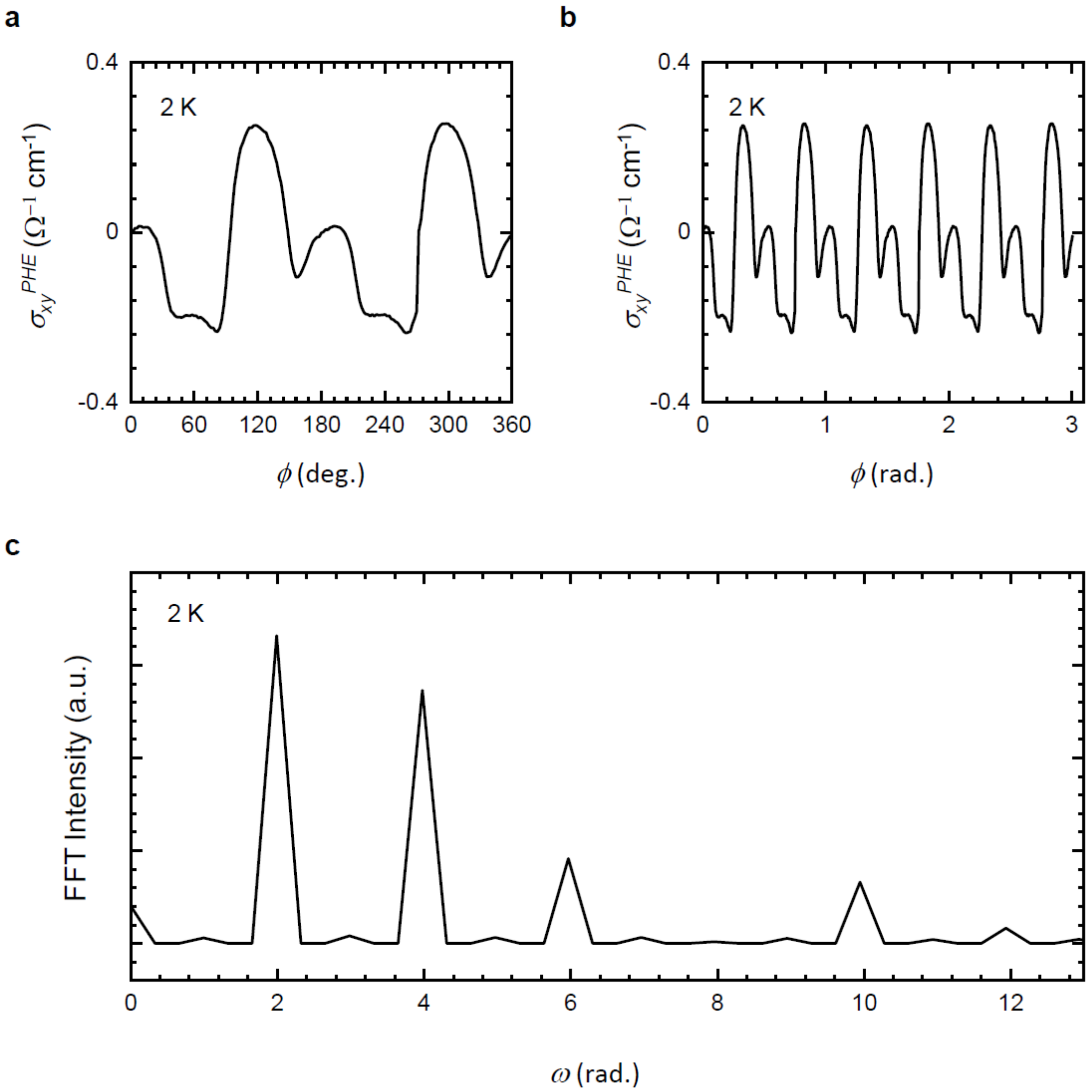

Figure s4 Process of fast Fourier transform of planar Hall effect curves. To see the dependency on each harmonic in the planar Hall effect curves, the fast Fourier transform was performed. **a.** The $T \sim 2$ K data of the planar Hall effect are shown to demonstrate the process of performing fast Fourier transforms in this work. **b.** The plot of the planar Hall effect was converted into radians. **c.** The plot of the performed fast Fourier transform.

The fast Fourier transform (FFT) was performed to observe the ω dependence of the $\sigma_{xy}^{PHE}(\phi)$ curves below 30 K. As an example, to demonstrate the FFT process, the 2 K $\sigma_{xy}^{PHE}(\phi)$ curve is used (Fig. s4a). Then, we converted ϕ into radians, and repetition of the

$\sigma_{xy}^{PHE}(\phi)$ curve was performed to increase the accuracy of the FFT data (Fig. s4b). Then, the FFT was performed by using the FFT function in the Origin program.

Note 3. Theoretical derivation of orthogonal magnetization from magnetic octupole ordering and its effect on the planar Hall effect.

Results from the Hubbard model for pyrochlore iridates

The Hubbard model for pyrochlore iridates is

$$H = H_0 + H_U + H_B, \tag{2}$$

where H_0 is the hopping Hamiltonian, H_U is the Hubbard repulsion, and H_B is the Zeeman term. Specifically,

$$\begin{aligned} \mathrm{H}_0 &= \sum_{\langle ij \rangle} c_{i\alpha}^{+} \alpha_{ij}(\delta)\big(t_1 + i t_2 \overrightarrow{d_{ij}} \times \vec{\sigma}_{\alpha\beta}\big) c_{j\beta} \\ &\quad + \sum_{\langle\langle ij \rangle\rangle} c_{i\alpha}^{+}\big(t_3 + i\big(t_4 \overrightarrow{R_{ij}} + t_5 \overrightarrow{D_{ij}}\big) \times \vec{\sigma}_{\alpha\beta}\big) c_{j\beta}, \\ \mathrm{H}_\mathrm{U} &= U \sum_i n_{i\uparrow} n_{i\downarrow}, \\ \mathrm{H}_\mathrm{B} &= -\vec{B} \cdot \sum_i c_{i\alpha}^{+} \vec{\sigma}_{\alpha\beta} c_{i\beta}, \end{aligned} \tag{3}$$

where $\mathrm{t}_{1,2,3,4,5}$ are the hopping parameters as a function of super-exchange hopping $\mathrm{t}_{\mathrm{oxy}}$, $\alpha_{ij}(\delta)$ is the change in the hopping parameters induced by strain, $\overrightarrow{d_{ij}}$ $\big(\overrightarrow{R_{ij}}, \overrightarrow{D_{ij}}\big)$ are the nearest (next-nearest) Dzyaloshinskii-Moriya (DM) vectors, and $\vec{\sigma}$ is the vector of Pauli matrices. The hopping parameters are defined as

$$
\begin{aligned}
t_1 &= \frac{130}{243} + \frac{17}{324} t_\sigma - \frac{79}{243} t_\pi, \\
\mathrm{t}_2 &= \frac{28}{243} + \frac{15}{243} t_\sigma - \frac{40}{243} t_\pi, \\
\mathrm{t}_3 &= \frac{233}{2916} t'_\sigma - \frac{407}{2187} t'_\pi, \\
\mathrm{t}_4 &= \frac{t'_\sigma}{1458} + \frac{220}{2187} t'_\pi, \\
\mathrm{t}_5 &= \frac{25}{1458} t'_\sigma + \frac{460}{2187} t'_\pi.
\end{aligned}
\tag{4}
$$

where $\mathrm{t}_\sigma = -0.8 t_{oxy}, t_\pi = -\frac{2}{3} t_\sigma, t'_\sigma = 0.08 t_\sigma,$ and $t'_\pi = 0.08\, t_\pi$. We set $\mathrm{t}_{\mathrm{oxy}} = 1$. The change in the hopping parameters is defined as

$$
\alpha_{ij}(\delta) = \begin{cases} 1 - \delta\ (\mathrm{ij} = 12,13,14). \\ 1 + \delta\ (ij = 23,24,34). \end{cases} \tag{5}
$$

The DM vectors are defined as

$$
\begin{aligned}
\overrightarrow{d_{ij}} &= 2\overrightarrow{f_{ij}} \times \vec{x}_{ij}, \\
\overrightarrow{R_{ij}} &= \vec{x}_{ik} \times \vec{x}_{kj}, \\
\overrightarrow{D_{ij}} &= \overrightarrow{d_{ik}} \times \overrightarrow{d_{kj}},
\end{aligned}
\tag{6}
$$

where $\overrightarrow{x_{ij}} = \overrightarrow{x_j} - \overrightarrow{x_i}$ is the displacement between sites *i* and *j* and $\overrightarrow{f_{ij}}$ is the vector from the unit cell center to the bond center between sites *i* and *j*.

The Hubbard repulsion is expressed in the mean-field approximation as

$$
\mathrm{H_U} \approx -U \sum_i \left(2\vec{J_i} \cdot \langle \vec{J_i} \rangle - \langle \vec{J_i} \rangle^2 \right), \tag{7}
$$

where $\vec{\mathrm{J}}_\mathrm{i} = \frac{1}{2} \sum_{\alpha\beta} c^+_{i\alpha} \vec{\sigma}_{\alpha\beta} c_{i\beta}$ is the spin operator. We set $\mathrm{U} = 1.485 t_{oxy}$ or $\mathrm{U} = 1.5 t_{oxy}$. Lastly, the magnetic field is applied in either the $[111]$ plane or the $[11\bar{2}]$ plane, That is,

$$\vec{B} = B\left(\frac{(1,-1,0)}{\sqrt{2}}\cos\phi + \frac{(1,1,-2)}{\sqrt{6}}\sin\phi\right),$$
$$\vec{B} = B\left(\frac{(1,-1,0)}{\sqrt{2}}\cos\theta + \frac{(1,1,1)}{\sqrt{3}}\sin\theta\right). \tag{8}$$

δ and B are variables. In the rest of this part, we change the basis to $\hat{x} \parallel [1\bar{1}0], \hat{y} \parallel [11\bar{2}]$, and $\hat{z} \parallel [111]$. Using the model, we self-consistently calculate the ground state of the Hubbard model with a $32 \times 32 \times 32$ *k*-mesh. The results are shown in Figs. s5. The blue solid line corresponds to the A_2-octupole, the orange dotted line to the T_{1x}-octupole, the orange circles to the T_{1y}-octupole, and the orange solid line to the T_{1z}-octupole. The definition of each multipole is

$$\begin{aligned}
A_2 &= \frac{1}{4\sqrt{3}}\langle\sigma_{1x} + \sigma_{1y} + \sigma_{1z} + \sigma_{2x} - \sigma_{2y} - \sigma_{2z} - \sigma_{3x} + \sigma_{3y} - \sigma_{3z} - \sigma_{4x} \\
&\qquad - \sigma_{4y} + \sigma_{4z}\rangle, \\
T_{1x,o} &= \frac{1}{4\sqrt{2}}\langle-\sigma_{1y} - \sigma_{1z} + \sigma_{2y} + \sigma_{2z} + \sigma_{3y} - \sigma_{3z} - \sigma_{4y} + \sigma_{4z}\rangle, \\
T_{1y,o} &= \frac{1}{4\sqrt{3}}\langle-\sigma_{1x} - \sigma_{1z} + \sigma_{2x} - \sigma_{2z} + \sigma_{3x} + \sigma_{3z} - \sigma_{4x} + \sigma_{4z}\rangle, \\
T_{1z,o} &= \frac{1}{4\sqrt{3}}\langle-\sigma_{1x} - \sigma_{1y} + \sigma_{2x} - \sigma_{2y} - \sigma_{3x} + \sigma_{3y} + \sigma_{4x} + \sigma_{4y}\rangle,
\end{aligned} \tag{9}$$

where $\langle \dots \rangle$ is the expectation value. Since we change the basis, the T_1-octupoles transform as

$$\begin{aligned}
T_{1x} &= \frac{T_{1x,o} - T_{1y,o}}{\sqrt{2}}, \\
T_{1y} &= \frac{T_{1x,o} + T_{1y,o} - 2T_{1z,o}}{\sqrt{6}}, \\
T_{1z} &= \frac{T_{1x,o} + T_{1y,o} + T_{1z,o}}{\sqrt{3}},
\end{aligned} \tag{10}$$

while A_2-octupole does not transform. Magnetization is defined as $\mathrm{M_a} = \sum_i \langle\sigma_{ia}\rangle$.

In Fig. s5, we show the emergence of T_1-octupoles under strain and a magnetic field.

The ground state without δ and B is all-in-all-out (AIAO), which carries only the A_2-octupole. When δ varies from 0 to 0.01 without B, T_{1z} appears and is proportional to δ. (See Fig. s5a.) Furthermore, when B varies from 0 to 0.02 with $\delta = 0.005$, T_{1x} and T_{1y} appear. (See Fig. s5b.) T_{1x} is proportional to B, while T_{1y} is proportional to B^2. Hence, the interplay of strain and the magnetic field induces T_1-octupoles.

In Fig. s6, we show the angular dependence of A_2- and T_1-octupoles. First, let us consider $\vec{B}$ in the $[111]$ plane. When ϕ varies from 0 to 2π with $\delta = 0.005$, $U = 1.485t_{oxy}$, and $B = 0.02$ and $B = 0.02$, each order parameter has a distinct angular dependence. The A_2-octupole and T_{1z}-octupole have a $\sin 3\phi$ dependence, as shown in Fig. s6a. This is explained by the threefold rotation about the *z*-axis of the strained crystal. However, the T_{1x}-octupole and T_{1y}-octupole have $\cos\phi$ and $\sin\phi$ dependences, respectively. Next, let us consider $\vec{B}$ in the $[11\bar{2}]$ plane. When θ varies from 0 to 2π with $\delta = 0.01, U = 1.500t_{oxy}$, and $B = 0.02$, the order parameters show different angular dependence from above. A_2-octupole and T_{1z}-octupole depend on $\sin\theta$, while T_{1x} and T_{1y}-octupoles depend on $\cos\theta$ and $\cos 2\theta$, respectively. Since these T_1-octupoles have the same symmetry as the dipolar order or magnetization, T_{1x} and T_{1y} have the same angular dependence as the induced magnetization.

Orthogonal magnetization from the A_2-octupole and T_1-octupole

Although the A_2-octupole and T_1-octupole are magnetic octupoles, they are different for two reasons. The first reason is that the magnetic point groups of the A_2-octupole and T_1-octupole are different, $-4'3m'$ and $-42'm'$, respectively. $-4'3m'$ carries 3-fold rotation symmetries, while $-42'm'$ does not. The second reason is that the A_2-octupole is a scalar,

while the T1-octupole is a vector. Whether the order parameter is a scalar or vector changes how the magnetic field and octupoles are coupled to each other. Specifically, the free energy under a magnetic field $\vec{H}$ and A2-octupole A_2 is given by

$$\mathrm{F}_{\mathrm{A2}} = -A_2 H_x H_y H_z, \tag{11}$$

while the free energy under a magnetic field and T1-octupole $\overrightarrow{\mathrm{T}_1}$ is given by

$$\mathrm{F}_{\mathrm{T1}} = -\overrightarrow{T_1} \cdot \vec{P}(\vec{H}), \tag{12}$$

where

$$P_i(\vec{H}) = -3H_i^3 + \frac{9}{2} H_i (H_j^2 + H_k^2). \tag{13}$$

Here, i, j, k is a cyclic permutation of x, y, z. Magnetization is the derivative of the free energy induced by a magnetic field. From the free energy of the A2-octupole, we obtain

$$M'_{A_2,i} = A_2 H_j H_k, \tag{14}$$

and from the free energy of the T1-octupole, we obtain

$$\begin{aligned} M'_{T_1,i} = T_{1i}\left(-9H_i^2 + \frac{9}{2}(H_j^2 + H_k^2)\right) + T_{1j}(9H_i H_j) \\ + T_{1k}(9H_i H_k). \end{aligned} \tag{15}$$

The components perpendicular to the magnetic field here are called orthogonal magnetization. Please note that this theory is for the small field limit.

From here, we change the basis to $\hat{x} \parallel [1\bar{1}0], \hat{y} \parallel [11\bar{2}]$, and $\hat{z} \parallel [111]$ for both the T1-octupole and magnetic field. The magnetic field is $\vec{\mathrm{H}} = \mathrm{H}(\cos\phi, \sin\phi, 0)$ in our physical situation. First, let us consider the magnetic field is in the *xy*-plane, i.e., $\vec{\mathrm{H}} =$

$\mathrm{H}(\cos\phi\,,\sin\phi\,,0)$. On this basis, the magnetization from the A$_2$-octupole is

$$M_{A_2,x} = -\frac{A_2}{\sqrt{6}}H^2\sin 2\phi\,,$$
$$M_{A_2,y} = -\frac{A_2}{\sqrt{6}}H^2\cos 2\phi\,, \tag{16}$$
$$M_{A_2,z} = -\frac{A_2}{2\sqrt{3}}H^2,$$

and that from the T$_1$-octupole is

$$\begin{aligned} M_{T_1,x} &= -\frac{3}{4}H^2\big(2\,T_{1x} + T_{1x}\cos 2\phi \\ &\quad + \big(-5\sqrt{2}T_{1z} + T_{1y}\big)\sin 2\phi\big), \\ M_{T_1,y} &= \frac{3}{4}H^2\big(-2\,T_{1y} + \big(5\sqrt{2}\,T_{1z} + T_{1y}\big)\cos 2\phi \\ &\quad - T_{1x}\sin 2\phi\big), \\ M_{T_1,z} &= \frac{3}{4}H^2\big(2\,T_{1z} + 5\sqrt{2}\,T_{1y}\cos 2\phi \\ &\quad + 5\sqrt{2}\,T_{1x}\sin 2\phi\big). \end{aligned} \tag{17}$$

Next, let us consider the magnetic field is in the xz-plane, i.e., $\vec{\mathrm{H}} = \mathrm{H}(\cos\theta\,,0,\sin\theta)$. The A$_2$-octupole gives

$$M_{A_2,x} = -\frac{A_2}{2\sqrt{3}}H^2\sin 2\theta\,,$$
$$M_{A_2,y} = \frac{A_2}{\sqrt{6}}H^2\cos^2\theta\,, \tag{16}$$
$$M_{A_2,z} = -\frac{A_2}{4\sqrt{3}}H^2(-1 + 3\cos 2\theta),$$

and T$_1$-octupole gives

$$
\begin{aligned}
M_{T_1,x} &= \frac{3}{8} H^2 \big(T_{1x}(-1 + 7\cos 2\theta) \\
&\quad - 2\big(5\sqrt{2} T_{1y} + 4 T_{1z}\big) \sin 2\theta \big), \\
M_{T_1,y} &= \frac{3}{8} H^2 \Big((-3 + 5\cos 2\theta)\, T_{1y} \\
&\quad - 5\sqrt{2}\,(1 + \cos 2\theta) T_{1z} \\
&\quad - 10\sqrt{2} T_{1x} \sin 2\theta \Big), \\
M_{T_1,z} &= -\frac{3}{4} H^2 \big(-8\, T_{1z} \sin^2\theta + 4 T_{1x} \sin 2\theta \\
&\quad + \big(5\sqrt{2}\, T_{1y} + 4\, T_{1z}\big) \cos^2\theta \big).
\end{aligned}
\tag{17}
$$

Planar Hall conductivity from the A_2-octupole and T_1-octupole

The Onsager relation for the Hall conductivity is $\sigma_{ij}(\vec{H},\vec{M}) = \sigma_{ji}(-\vec{H},-\vec{M})$. The planar Hall conductivity is defined as the symmetric part of the Hall conductivity ($\sigma_{ij}(\vec{H},\vec{M}) = \sigma_{ji}(\vec{H},\vec{M})$). Specifically, the planar Hall conductivity (PHC) is

$$\sigma_{ij}^{P} = \sigma_0(H_iH_j) + \sigma_1(M_iH_j + M_jH_i) + \sigma_2(M_iM_j). \quad (18)$$

σ_0, σ_1, and σ_2 are constants.

Here, we change the basis to $\hat{x} \parallel [1\bar{1}0], \hat{y} \parallel [11\bar{2}]$, and $\hat{z} \parallel [111]$. Let us consider the magnetic field in the [111] plane, $\vec{\mathrm{H}} = \mathrm{H}(\cos\phi, \sin\phi, 0)$ The longitudinal magnetization (dipole) $\vec{M_D} = \chi\vec{H}$, and orthogonal magnetization $\overrightarrow{M_{A_2}}$ and $\overrightarrow{M_{T_1}}$. Note that the angular dependences of octupoles are $\mathrm{A}_2 \propto \sin 3\phi$, $T_{1x} \propto \cos\phi$, $T_{1y} \propto \sin\phi$, and $T_{1z} \propto \sin 3\phi$ from the Hubbard model.

We divide the PHC on the *xy*-plane σ_{xy}^{P} into three parts according to the origin. First, the PHC from $\vec{H}$ and $\overrightarrow{M_D}$ is given by

$$\sigma_{xy}^{P,M_D} \propto H^2 \sin 2\phi. \quad (19)$$

Second, the PHC from $\overrightarrow{M_{A_2}}$ and $\mathrm{A}_2 \propto \sin 3\phi$ is given by

$$\begin{aligned} \sigma_{\mathrm{xy}}^{P,A_2} = {} & H^3(a_1\cos\phi + a_2\sin 2\phi + a_3\sin 4\phi) \\ & + H^4(b_1\sin 2\phi + b_2\sin 4\phi \\ & + b_3\sin 10\phi), \end{aligned} \quad (20)$$

where a_i and b_i are constants. Third, the PHC from $\overrightarrow{M_{T_1}}$, $T_{1x} \propto \cos\phi$, $T_{1y} \propto \sin\phi$, and

$T_{1z} \propto C + D\sin 3\phi$ is given by

$$\sigma_{\mathrm{xy}}^{P,T_1} = H^3(c_1\cos\phi + c_2\sin 2\phi + c_3\sin 4\phi) + H^4(d_1\cos\phi + d_2\sin 2\phi + d_3\sin 4\phi + d_4\sin 6\phi + d_5\sin 10\phi), \tag{21}$$

where c_i and d_i are constants. Here, we note some points. First, $\sin 2\phi$ is dominated by σ_{xy}^{P,M_D} since it is proportional to the leading order H^2. Second, $\sin 4\phi$ is dominated by σ_{xy}^{P,A_2} since the A_2-octupole is the largest among all the order parameters. Third, $\sin 6\phi$ appears only in σ_{xy}^{P,T_1}. Hence, we denote $\sin 2\phi$ as the dipole term, $\sin 4\phi$ as the A_2-octupole term, and $\sin 6\phi$ as the T_1-octupole term.

$$\begin{aligned}\sigma_{\mathrm{xy}}^{P,A_2} &= H^3(a_2\sin 2\phi + a_3\sin 4\phi) + H^4(b_1\sin 2\phi + b_2\sin 4\phi + b_3\sin 10\phi),\\ \sigma_{\mathrm{xy}}^{P,T_1} &= H^3(c_2\sin 2\phi + c_3\sin 4\phi) + H^4(d_2\sin 2\phi + d_3\sin 4\phi + d_4\sin 6\phi + d_5\sin 10\phi).\end{aligned} \tag{22}$$

Using equations (19) and (22), we fitted the experimental planar Hall conductivity (Fig.s8).

We calculated the magnetization induced by strain and magnetic field in the octupolar system $Nd_2Ir_2O_7$ (Fig. s5). We recall that the planar Hall conductivity in $Nd_2Ir_2O_7$ is generated by the orthogonal magnetization that results from the coupling between the octopolar orders (higher-rank multipoles) and the magnetic field. Since orthogonal magnetization is the

coupling between the higher rank multipoles and magnetic field, it should have small values. As expected (Fig. s5c and 5b), the magnetization (orthogonal magnetization) induced by the magnetic field in the strained $Nd_2Ir_2O_7$ is very small (at the scale of 10^{-3} μ_B/atom). Hence, the octupolar contributions to the planar Hall conductivity are small.

Longitudinal magnetoconductivity from A_2 and T_1-octupoles

Meanwhile, we use Onsager's relation for longitudinal conductivity as well.

$$\sigma_{xx} = \sigma_0(H_x^2) + \sigma_1(M_x H_x) + \sigma_2(M_x^2). \tag{23}$$

First, let us consider the magnetic field in the [111] plane, $\vec{\mathrm{H}} = \mathrm{H}(\cos\phi, \sin\phi, 0)$, The obtained conductivity is anisotropic magnetoconductivity (AMC). The first term is

$$\sigma_{xx}^{1} \propto \cos 2\phi, \tag{24}$$

$\overrightarrow{M_{A_2}}$ from A_2-octupole gives rise to

$$\begin{aligned}\sigma_{xx}^{\mathrm{A}_2} &= H^3(e_1 \sin\phi + e_2 \sin 3\phi) \\ &+ H^4(f_1 \cos 2\phi + f_2 \cos 4\phi + f_3 \cos 6\phi \\ &+ f_4 \cos 10\phi),\end{aligned} \tag{25}$$

and $\overrightarrow{M_{T_1}}$ from T_1-octupole gives rise to

$$\begin{aligned}\sigma_{xx}^{\mathrm{T}_1} &= H^3(g_1 \sin\phi + g_2 \sin 3\phi + g_3 \cos 2\phi \\ &+ g_4 \cos 4\phi + g_5 \cos 6\phi) \\ &+ H^4(h_1 \sin\phi + h_2 \sin 3\phi + h_3 \cos 2\phi \\ &+ h_4 \cos 4\phi + h_5 \cos 6\phi \\ &+ h_6 \cos 8\phi + h_7 \cos 10\phi).\end{aligned} \tag{26}$$

The experimental AMC for the measured temperature of $Nd_2Ir_2O_7$ film and the fitting result is shown in Fig. s9.

Next, let us consider the magnetic field in the $[11\bar{2}]$ plane, $\vec{\mathrm{H}} = \mathrm{H}(\cos\theta, 0, \sin\theta)$. The first term is

$$\sigma_{xx}^{1} \propto \cos 2\theta, \tag{24}$$

$\overrightarrow{M_{A_2}}$ from A_2-octupole and $\overrightarrow{M_{T_1}}$ from T_1-octupole give rise to

$$\begin{aligned}\sigma_{xx} = H^3(&x_1 \sin\theta + x_2 \sin 3\theta + x_3 \sin 4\theta + x_4 \sin 5\phi \\ &+ x_5 \cos 2\theta + x_6 \cos 4\theta) \\ +H^4(&y_1 \sin\theta + y_2 \sin 3\theta + y_3 \sin 5\phi + y_4 \cos 2\theta \\ &+ y_5 \cos 4\theta + y_6 \cos 6\theta).\end{aligned} \tag{25}$$

The experimental longitudinal magnetoconductivity with out-of-plane rotation for the measured temperature of $Nd_2Ir_2O_7$ film and the fitting result is shown in Fig. s10.

About the magnetic anisotropy of pyrochlore iridates

Ir spins have no magnetic anisotropy, although Ir is a d-electron system since Ir spins are considered spin-1/2. In the pyrochlore system, Ir ions are surrounded by an oxygen octahedron. Then, Ir d-orbitals are broken into t_{2g} and e_g orbitals by the crystal field. Moreover, Ir also has strong spin-orbit coupling, and t_{2g} is broken into $J = 1/2, 3/2$ orbitals. Because $\mathrm{J} = 1/2$ is half-filled at the Fermi level, Ir spins are considered spin-1/2. In contrast, Nd spins have magnetic anisotropy along the [111] and equivalent axes because they have a high spin $J_{Nd} = 9/2$. In our free energy model, since we consider the magnetic order of Ir spins only, the magnetic anisotropy seems to be obviated.

However, most transport phenomena in pyrochlore iridates come from Ir d electrons and not from Nd f electrons since f electrons are strongly localized near the ionic center. Thus, the magnetic anisotropy of Nd f electrons can indirectly affect the transport by changing the Ir spin configuration through the *f-d* exchange. Since we study the magnetic multipole-induced planar Hall effect, considering the Ir spin configuration in the free energy model is sufficient to include the effect of magnetic anisotropy of Nd spins.

Furthermore, magnetic multipoles have already been considered for magnetic anisotropy. Here, the magnetic multipoles are made up of spin clusters in the unit cell. We classify every possible spin configuration into magnetic multipoles by group theory. There are a total of 12 magnetic multipoles since we have 4 atoms in the 3-dimensional unit cell: 3 dipoles, an A_2-octupole, 3 T_1-octupoles, 3 T_2-octupoles, and 2 E-dotriacontapoles. Every possible magnetic order can be expressed as a linear combination of the 12 magnetic multipoles. Meanwhile, magnetic anisotropy affects the spin configuration, either by making spins point along their easy axes or through the *f-d* exchange. The associated spin configuration can be

described by the linear combination of magnetic multipoles. As the free energy contains the coupling of magnetic multipoles to the magnetic field, our model includes the effect of magnetic anisotropy.

Lastly, we consider the effect of compressive strain on magnetic anisotropy. Magnetic anisotropy strongly depends on the temperature. In $Nd_2Ir_2O_7$, Ir spins order at 30 K, while Nd spins order at 15 K. Magnetic anisotropy becomes effective below 15 K, at which Nd spins order. However, compressive strain contributes little to magnetic anisotropy. The two largest energy scales that determine the physics in pyrochlore iridates are the energy gap between the t_{2g} and e_g orbitals (- 2 eV) and the spin-orbit coupling (-0.4 eV), which are much larger than the bandwidth. A compressive strain of approximately 1 %, in contrast, changes the band energy by a few percent of the bandwidth. Hence, the strain does not significantly change the magnetic anisotropy.

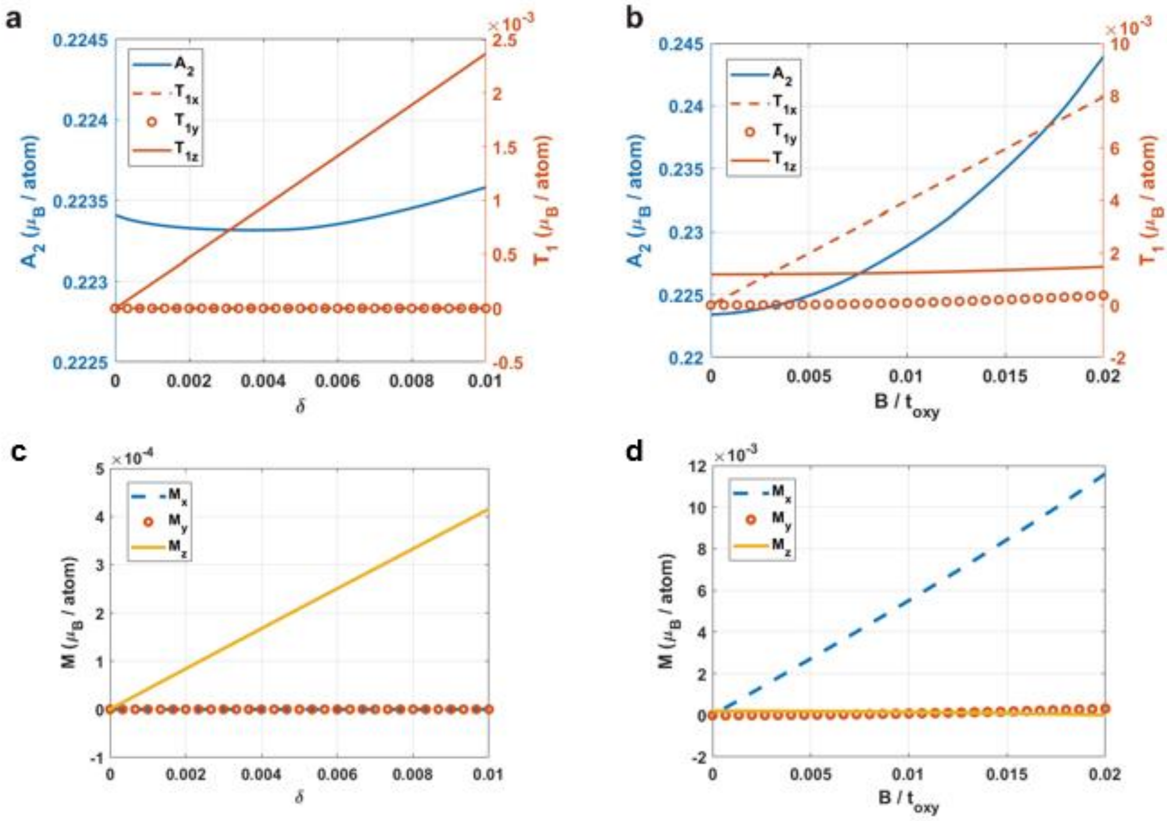

Figure s5 The emergence of T1-octupole under strain and magnetic field. We present A2-octupole and T1-octupoles under the change of strain and magnetic field. The blue solid line is A2, the orange dotted line is T_{1x}, the orange circles are T_{1y}, and the orange solid line is T_{1z}. **a.** The change of order parameters when the strain δ varies from 0 to 0.01 with $\vec{B} = 0$. When the strain is absent ($\delta = 0$), only A2-octupole exists. When $\delta \neq 0$, only T_{1z} emerges linearly to δ while T_{1x} and T_{1y} remains trivial. **b.** The change of order parameters when magnetic field strength varies from 0 to 0.02 with $\phi = 0, \delta = 0.005$. T_{1x} emerges linearly to δ, and other order parameters change quadratically to δ. **c.** The change of magnetization when the strain δ varies from 0 to 0.01 with $\vec{B} = 0$. **d.** The change of magnetization when magnetic field strength varies from 0 to 0.02 with $\phi = 0, \delta = 0.005$.

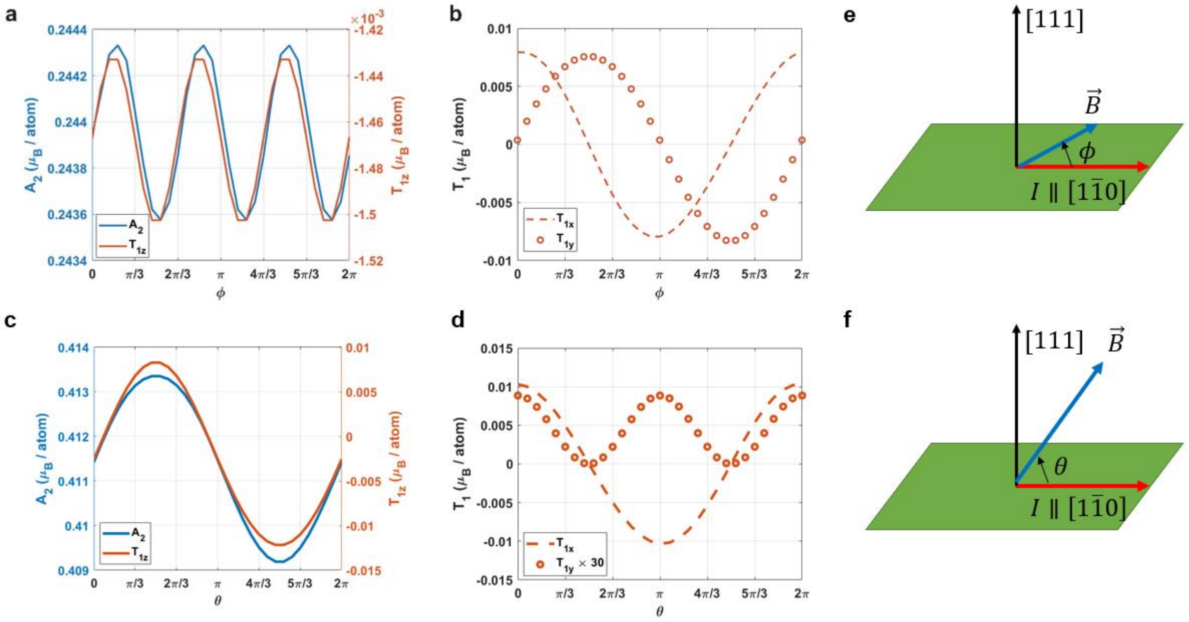

Figure s6 The angular dependence of A_2 and T_1-octupoles. We present the change of order parameters for two cases. a-b. $\vec{B}$ is in the $[111]$ plane. ϕ varies from 0 to 2π, with $\delta = 0.005, U = 1.485\, t_{oxy}$, and $B = 0.02$. **a.** A_2-octupole (blue solid line) and T_{1z}-octupole (orange solid line) which are proportional to $\sin 3\phi$. **b.** T_{1x} (orange dotted line) and T_{1y} (orange circles) which are proportional to $\cos\phi$ and $\sin\phi$, respectively. **c-d.** $\vec{B}$ is in the $[11\bar{2}]$ plane. θ varies from 0 to 2π, with $\delta = 0.01, U = 1.5\, t_{oxy}$, and $B = 0.02$. **c**. A_2-octupole (blue solid line) and T_{1z}-octupole (orange solid line) are proportional to $\sin 3\theta$. **d**. T_{1x} (orange dotted line) and 30 times T_{1y} (orange circles) which are proportional to $\cos\theta$ and $\cos 2\theta$, respectively. The schematics of the physical geometry when **e.** $\vec{B}$ in the $[111]$ plane and **f.** $\vec{B}$ in the $[11\bar{2}]$ plane.

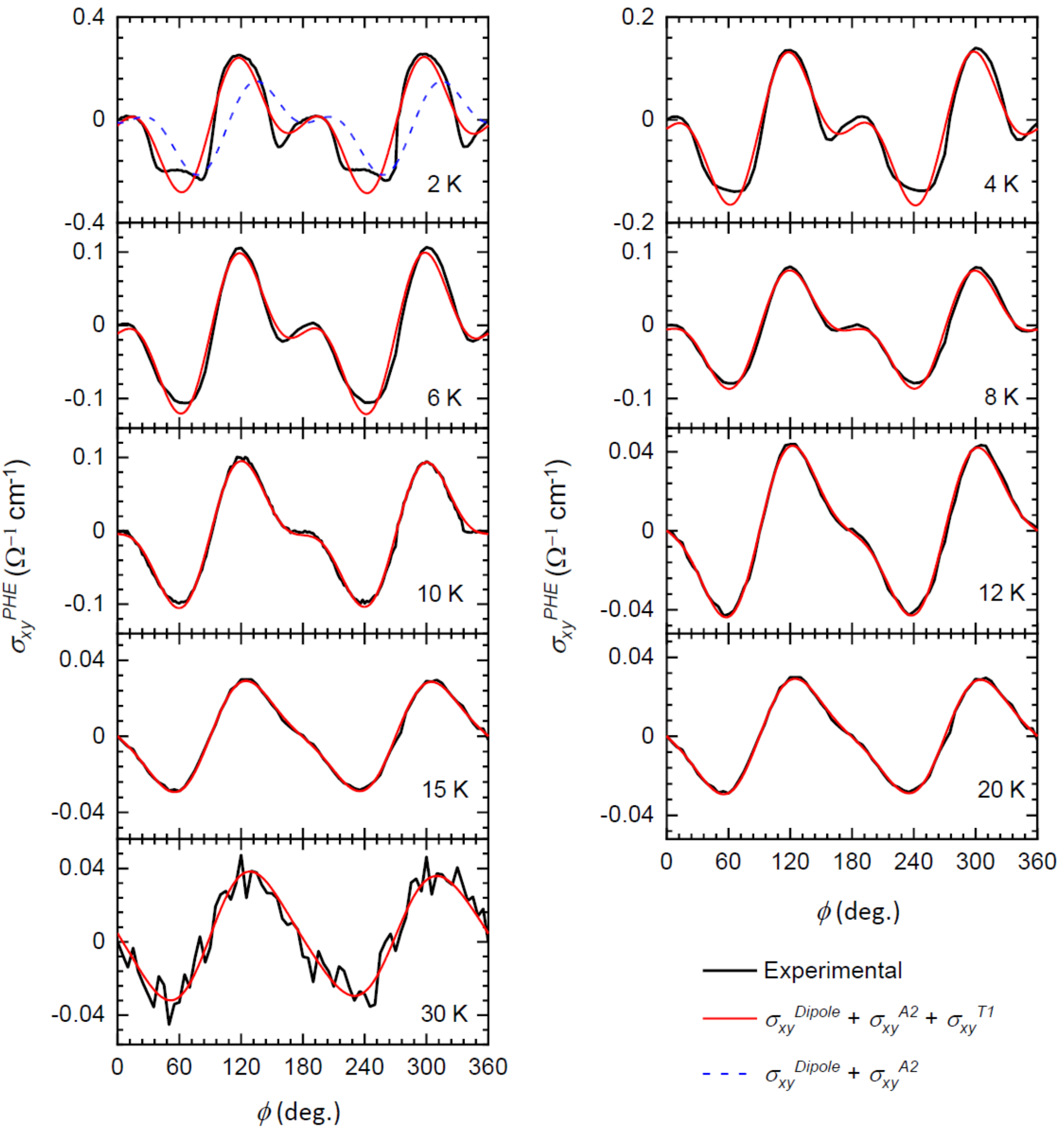

Figure s7 Plot of the calculated PHC below 30 K. All the displayed plots went through the same procedure as described in Fig. s3.

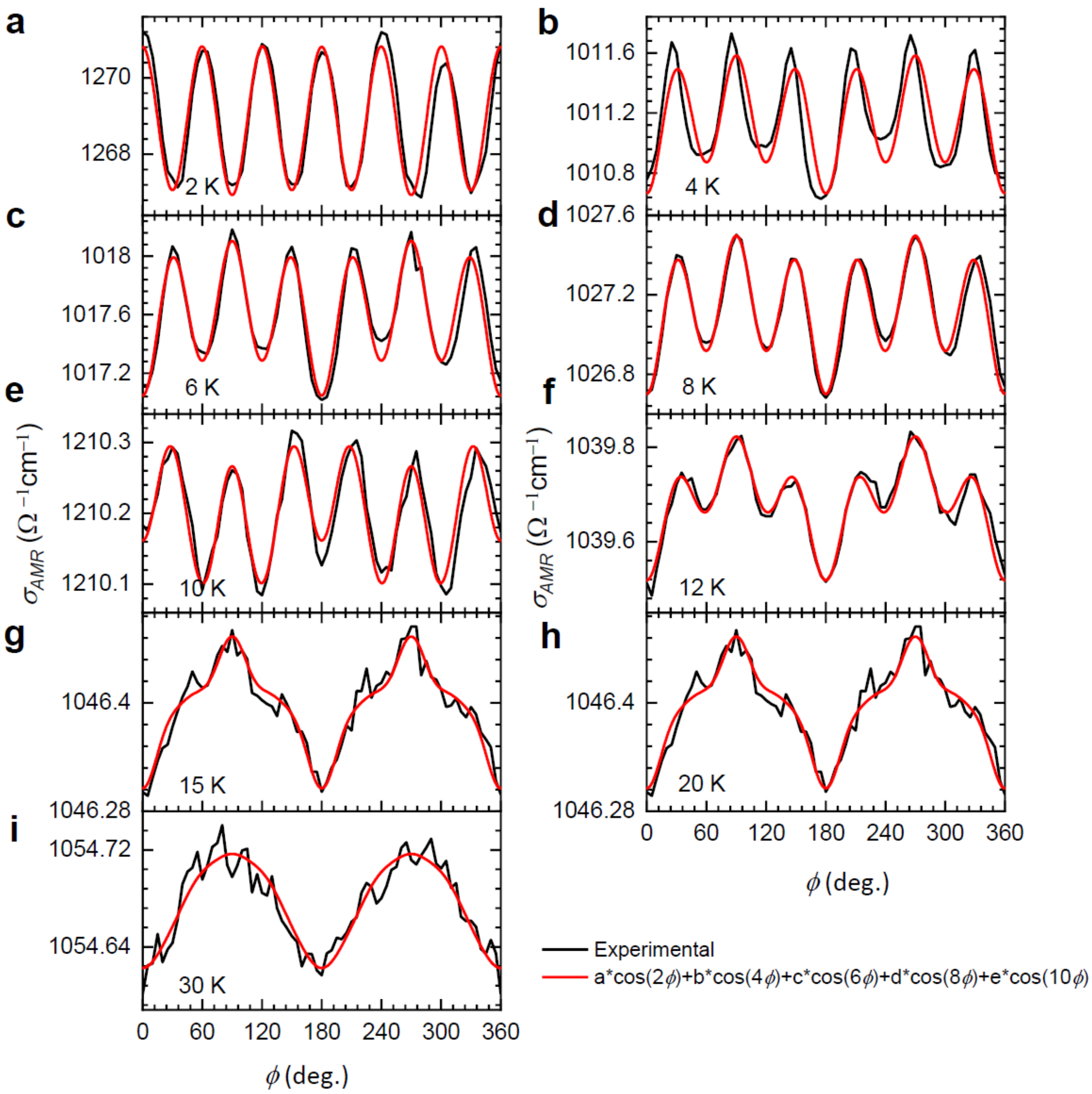

Figure s8 Anisotropic magnetoconductance and fitting with cluster multipoles contributions. σ_{AMR} (ϕ) measured at **a.** 2 K, **b.** 4 K, **c.** 6 K, **d.** 8 K, **e.** 10 K, **f.** 12 K, **g.** 15 K, **h.** 20, and **i.** 30 K. The black line is experimental data while the red line is the fitting result with theoretical AMR equation.

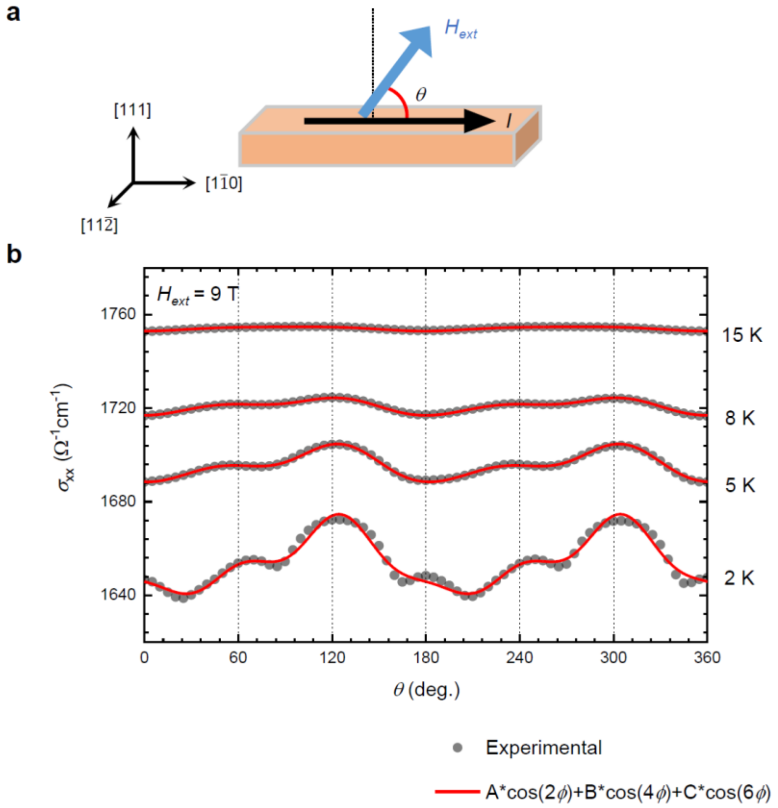

Figure s9 Temperature dependence of out-of-plane rotation magnetoconductivities. a. Schematic of out-of-plane rotation measurement. The current is applied along [1$\bar{1}$0] direction and magnetic field is rotated along [111] and [1 $\bar{1}$ 0] plane. **b.** Experimental magnetoconductivities below 15 K and their fitting.

Note 4. Anomalous planar Hall effect in the $Nd_2Ir_2O_7$ thin film.

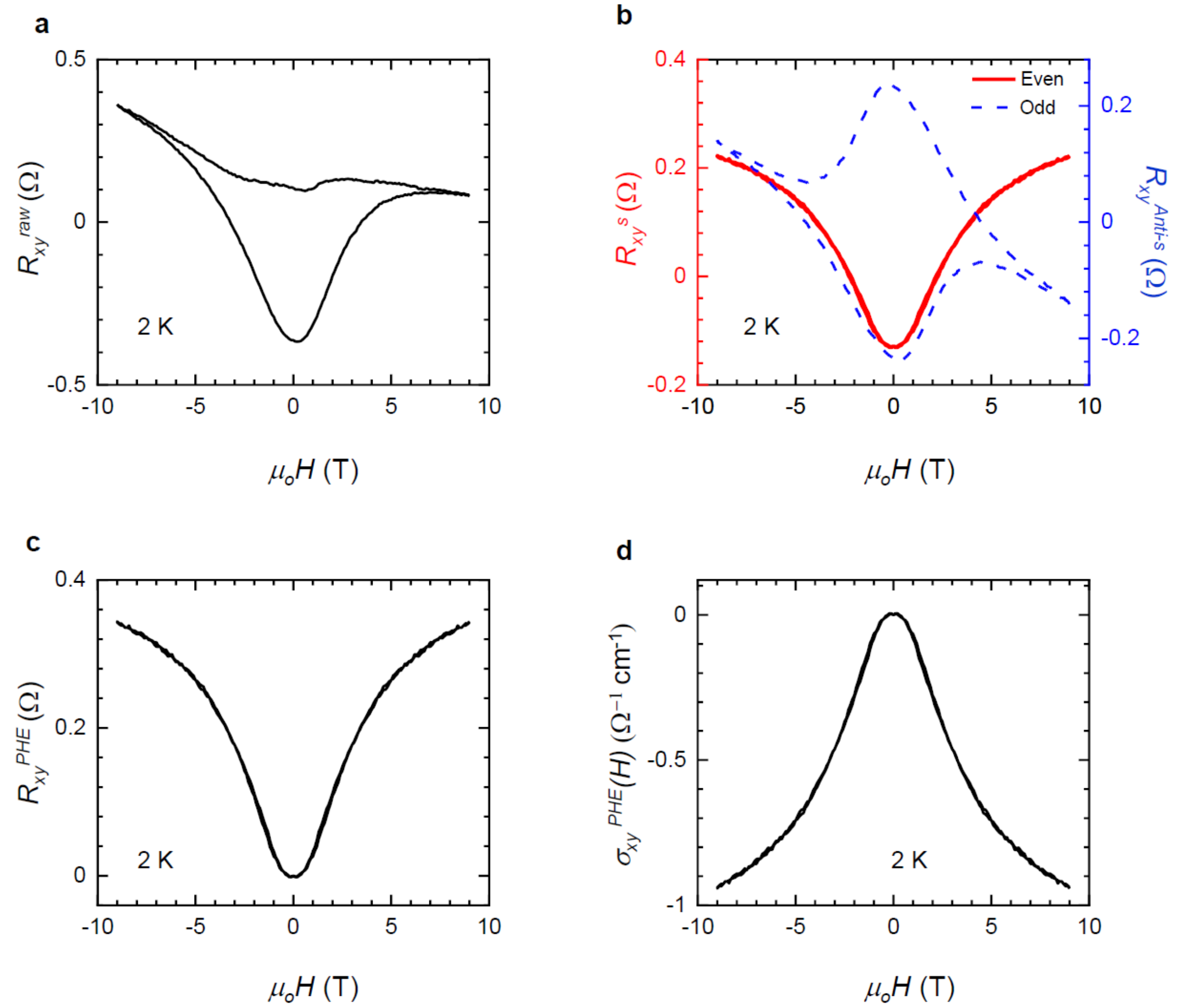

Figure s10 Symmetrization process of the planar Hall effect when the angle between H_{ext} and I is fixed. In this geometry, the angle between H_{ext} and I ($\pm$ 9 T) is fixed at 45°. The $T \sim 2$ K data are shown to demonstrate the process of extraction of the planar Hall effect in this geometry. **a.** The raw data of $R_{xy}{}^{raw}$ were measured at $\pm$ 9 T. **b.** The even (odd) part of the $R_{xy}{}^{raw}$ curve was extracted by the symmetrization (antisymmetrization) equation $R_{xy}^{s}(\phi, H) = \frac{R_{xy}(\phi,\ H) + R_{xy}(\phi,\ -H)}{2}$ ($R_{xy}^{Anti-s}(\phi, H) = \frac{R_{xy}(\phi = 0,\ H) - R_{xy}(\phi = 0,\ -H)}{2}$). **c.** Plot of the R_{xy}^{PHE} curve. The contribution of the longitudinal magnetoresistance was excluded by using equation (1). **d.** PHC $\sigma_{xy}^{PHE}(H)$ curve with respect to H obtained using $\sigma_{xy}^{PHE}(H) = \frac{-\rho_{xy}^{PHE}}{{\rho_{xy}^{PHE}}^2 + {\rho_{xx}}^2}$.

The same procedure as we used to calculate the $\sigma_{xy}^{PHE}(\phi)$ curves in Section 2 were applied to calculate the $\sigma_{xy}^{PHE}(H)$ curve.

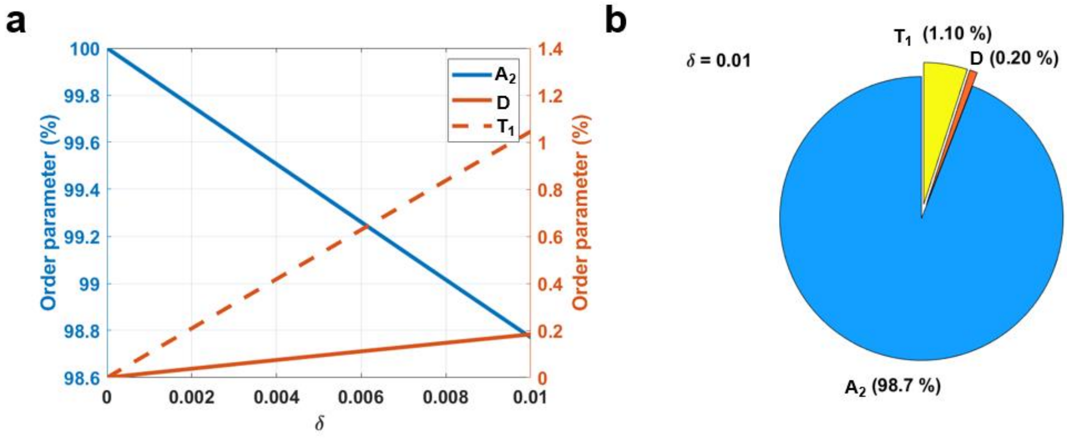

Figure s11 The proportions of cluster multipoles in strained $Nd_2Ir_2O_7$ film. a. The change in cluster multipoles by various strengths of strain δ. **b.** When strain is 1 % ($\delta = 0.01$), the proportion of cluster dipole (D), A_2 octupole, and T_1 octupole is shown.

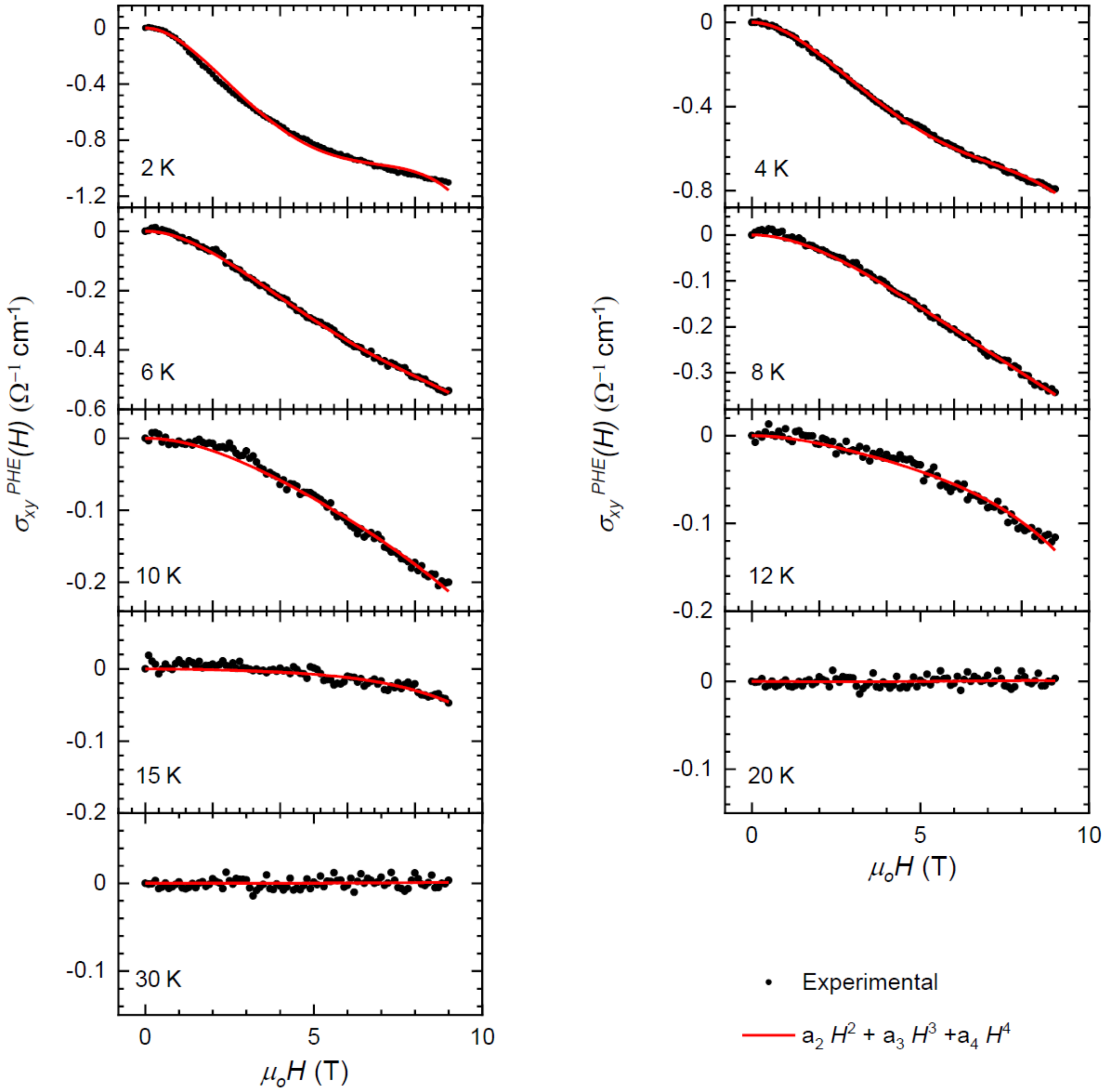

Figure s12 Fitting result of the measured *T*-dependent $\boldsymbol{\sigma_{xy}^{PHE}(H)}$ curve. All $\sigma_{xy}^{PHE}(H)$ curves were fitted with equations (19) and (22).

	Dipole	A_2-octupole	T_1-octupole
M	$\neq 0$	$= 0$	$= 0$
$\sigma_{xy}{}^{AHE}(H)$ (ref. 2)	$\neq 0$	$= 0$	$\neq 0$
$M_\perp$	$= 0$	$\neq 0$	$\neq 0$
$\sigma_{xy}{}^{PHE}(\phi)$	second	fourth	sixth
$\sigma_{xy}{}^{PHE}(H)$	$\sim H^2$	$\sim H^3$ and $\sim H^4$	$\sim H^3$ and $\sim H^4$

Table 1 Contributions of magnetic orderings to the anomalous and planar Hall effects. Each contribution of magnetic orderings to the anomalous and planar Hall effects is shown. The contribution of T_1-octupole ordering to the $\sigma_{xy}^{AHE}(H)$ of the $Nd_2Ir_2O_7$ thin film can induce a finite $\sigma_{xy}^{AHE}(H= 0)$[2]. In contrast, the orthogonal magnetization, $M_\perp$, is induced from cluster magnetic octupoles, affecting $\sigma_{xy}^{PHE}(\phi)$ and $\sigma_{xy}^{PHE}(H)$ with distinctive features.

References

1. Kim, W. J. et al. Unconventional anomalous Hall effect from antiferromagnetic domain walls of $Nd_2Ir_2O_7$ thin films. *Phys. Rev. B* **98,** 125103 (2018).

2. Kim, W. J. et al. Strain engineering of the magnetic multipole moments and anomalous Hall effect in pyrochlore iridate thin films. *Sci. adv.* **6** eabb1539 (2020).

3. Ge, J. et al. Unconventional Hall effect induced by Berry curvature. *National Sci. Rev.* **7**, 1879-1885 (2020).
